# Supplementary material for: Saccharomyces boulardii (CNCM I-745) improves intestinal damage in sepsis by remodeling intestinal flora structure
Source: Front Cell Infect Microbiol. 2025 Jul 31;15:1602792. doi: 10.3389/fcimb.2025.1602792 (PMC12350329; doi:10.3389/fcimb.2025.1602792)
Supplement: Supplementary file 1 [file DataSheet1.zip › summary/summary/6_Taxonomy_community/1_Taxonomy_profiling/2_Phylum/Group/Phylum_abund_top30.Group.stacked_bar.pdf]

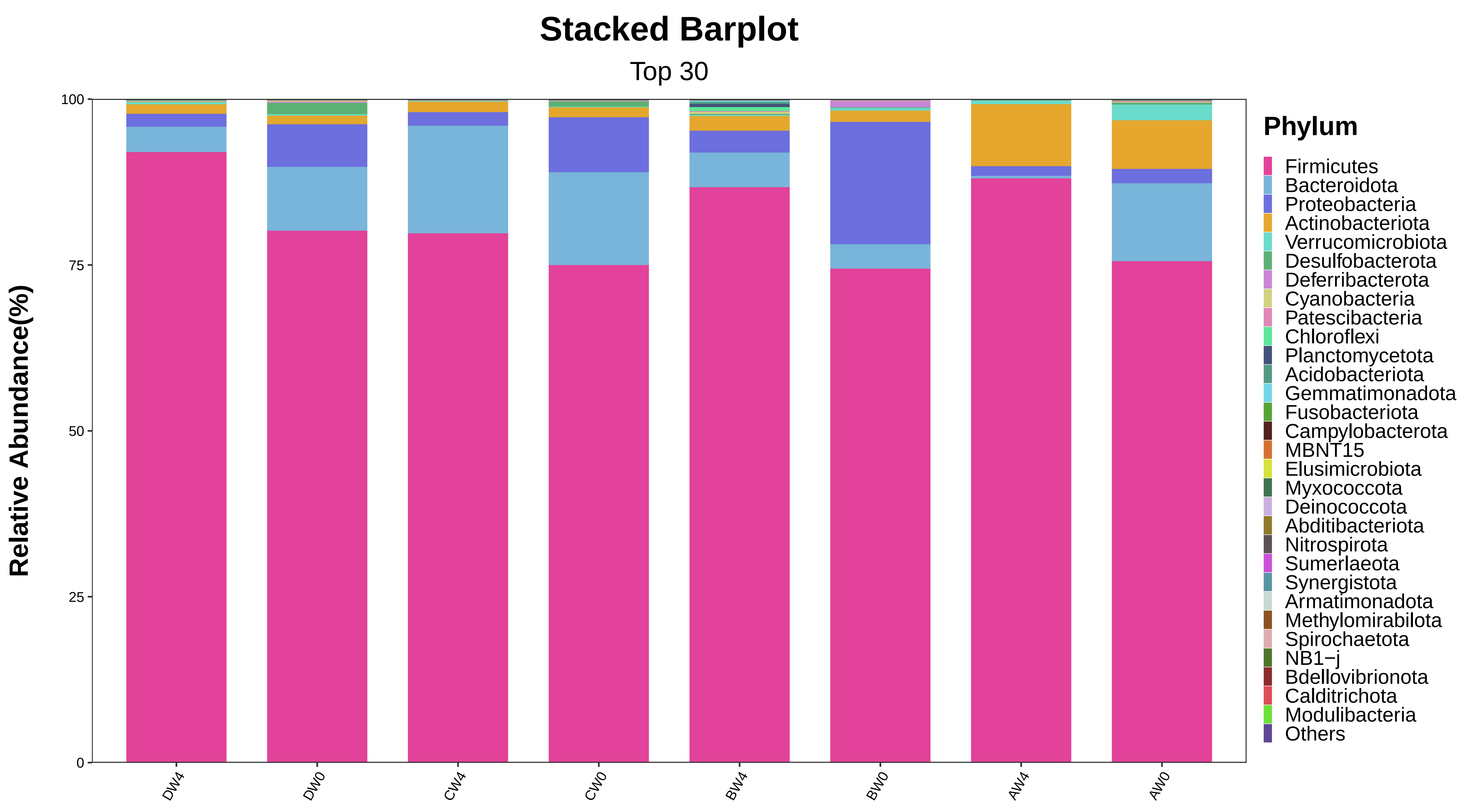

Stacked Barplot
Top 30
Relative Abundance(%)
100
75
50
25
0
DW4
DW0
CW4
CW0
BW4
BW0
AW4
AW0
Phylum
Firmicutes
Bacteroidota
Proteobacteria
Actinobacteriota
Verrucomicrobiota
Desulfobacterota
Deferribacterota
Cyanobacteria
Patescibacteria
Chloroflexi
Planctomycetota
Acidobacteriota
Gemmatimonadota
Fusobacteriota
Campylobacterota
MBNT15
Elusimicrobiota
Myxococcota
Deinococcota
Abditibacteriota
Nitrospirota
Sumerlaeota
Synergistota
Armatimonadota
Methylomirabilota
Spirochaetota
NB1−j
Bdellovibrionota
Calditrichota
Modulibacteria
Others
